# A case report of right coronary artery agenesis diagnosed by computed tomography coronary angiography

**DOI:** 10.1097/MD.0000000000019176

**Published:** 2020-02-14

**Authors:** Ernesto Forte, Bruna Punzo, Marco Agrusta, Marco Salvatore, Gianluca Spidalieri, Carlo Cavaliere

**Affiliations:** a IRCCS SDN, Naples; b Clinica Montevergine, Mercogliano, Italy.

**Keywords:** coronary agenesis, coronary anomalies, coronary computed tomography angiography, right coronary artery

## Abstract

**Introduction:**

Single coronary artery is a rare condition characterized by the origin of a coronary that supplies the entire heart from a single coronary ostium.

**Patient concerns:**

A 45-year-old woman with an altered exercise testing was addressed to a computed tomography coronary angiography (CTCA) to rule out coronary artery disease (CAD).

**Diagnosis:**

CTCA examination showed the absence of the right coronary artery (RCA). The left anterior descending artery and the left circumflex artery (LCX) presented regular origin and course and LCX provided the posterior interventricular artery and the posterolateral artery.

**Interventions:**

As CTCA highlighted the absence of potentially life-threatening features related to coronary anomaly, no surgical treatment was advised.

**Outcomes:**

The patient was dismissed, kept under pharmacological control and monitored over time.

**Conclusion:**

CTCA is the first-choice imaging modality in patients with ECG abnormalities properly allowing the differential diagnosis between CAD and congenital heart disease.

## 1 Introduction

Congenital agenesis of the right coronary artery (RCA) is a coronary anomaly characterized by a single coronary ostium from which the whole coronary tree takes origin. Coronary anomalies are often incidental findings in patients undergoing coronary angiography for coronary artery disease (CAD) exclusion. Cardiovascular computed tomography, thanks to its high spatial and contrast resolution, constitutes an excellent tool for the differential diagnosis between CAD and congenital anomalies providing typical findings.^[1]^

In this study, we report the case of a patient with congenital absence of RCA who performed a computed tomography coronary angiography (CTCA) for CAD suspicion.

## 2 Case report

An asymptomatic 45-year-old woman was referred to our institution with an exercise testing suggestive for myocardial ischemia (ST segment depression), according to AHA guidelines.^[2]^ She was not under pharmacological treatment. Echocardiography and biochemical parameters were within the normal limits. Afterwards, a CTCA was planned to rule out CAD. The examination was carried out by a 128-slice computed tomography (Revolution GSI, GE Healthcare) with 0.35 second rotation time, prospective ECG triggering and 40 mm total collimation width. The angiographic scan was preceded by a baseline acquisition for calcium score evaluation. Therefore, 100 mL of high concentration contrast medium (Iomeprol 400 mg I/mL, Iomeron 400, Bracco, Milan, Italy) were injected at 5 mL/s and bolus tracking technique was used to synchronize the start of the acquisition with contrast bolus arrival. Images were reconstructed using the GE advantage workstation 4.6 (GE Healthcare) and Cinematic Rendering Prototype (version 1.0.1, Syngo.VIA Frontier Platform, Siemens Healthineers).

**Figure 1:**
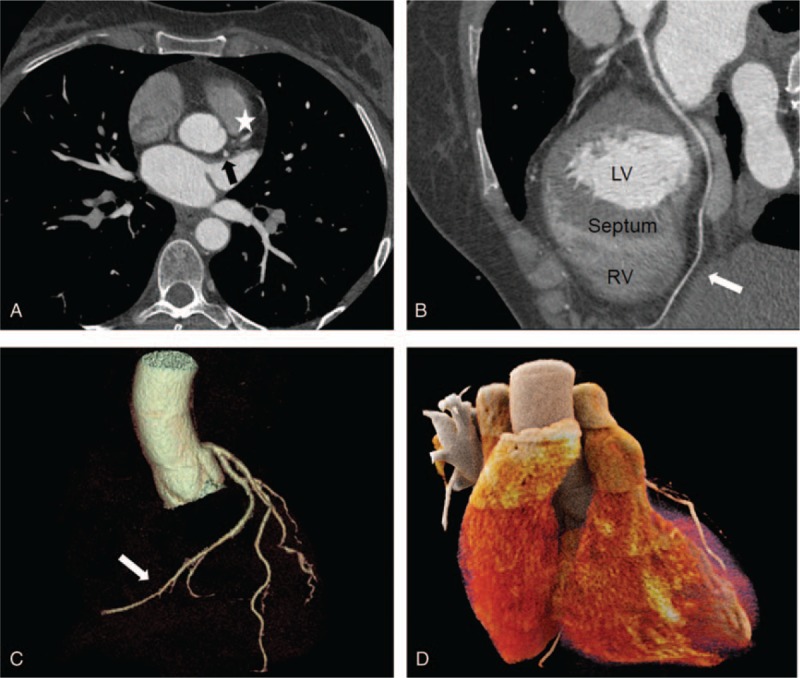
(A) Axial image showing an empty right coronary sinus while the left common trunk normally originates from the left coronary sinus giving rise to the LAD (white star) and the LCX (black arrow). (B and C) c-MPR and angiographic view reproducing LCX course: it runs in the left atrioventricular groove and supplies cardiac inferior wall, then it overtakes the crux cordis and reaches the acute margin of the heart (white arrow). (D) Cinematic rendering representing the absence of the RCA and the distal LCX. c-MPR = curved-multiplanar reformation, LAD = left anterior descending artery, LCX = left circumflex artery, LV = left ventricle, RCA = right coronary artery, RV = right ventricle.

**Figure 2:**
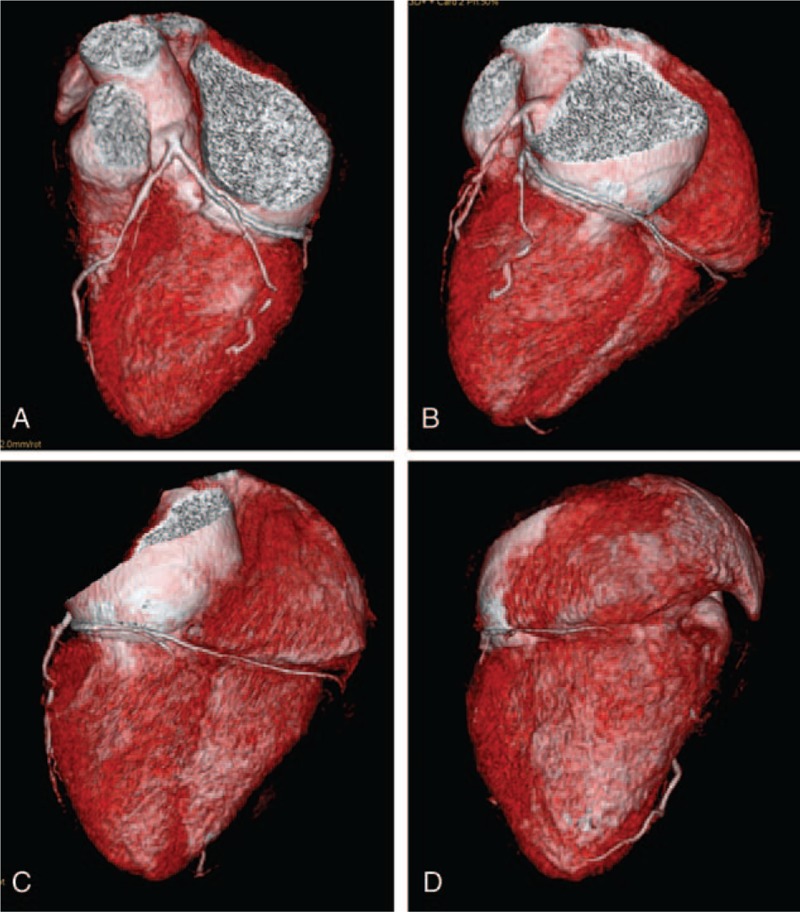
(A–D) 3D volume rendering images reproducing the course of the LCX. LCX = left circumflex artery.

The phase with least residual motion was utilized for further evaluation. Maximum intensity projection (MIP), multiplanar reformations (MPR), 3D volume rendering (VR) and cinematic rendering (CR) were generated (Figs. 1 and 2).

The examination showed the absence of any artery arising from the right sinus of Valsalva. The left anterior descending artery (LAD) and the left circumflex artery (LCX) presented regular origin and course with no evidence of CAD (Calcium score = 0). LCX provided a wide obtuse marginal branch, a filiform posterior interventricular artery and a noticeable posterolateral artery running in the inferior atrioventricular groove reaching the crux cordis up to the right atrioventricular sulcus. The LAD provided an acute margin branch and the conus artery while the sinoatrial nodal artery originated from proximal LCX. In light of imaging findings, the surgical intervention was not kept under consideration. Therefore, the patient was kept under pharmacological control by anti-platelets and monitored over time without any surgical intervention. Moreover, she was advised to avoid heavy exercise and to observe a proper nutrition and a healthy lifestyle to prevent the onset of coronary atherosclerosis.

## 3 Discussion

Single coronary artery belongs to the broad spectrum of coronary artery anomalies and constitutes a rare condition where the entire coronary arterial system arises from a solitary ostium.^[3]^ For single coronary artery anomalies, the Lipton classification, revised by Yamanaka and colleagues,^[4]^ is commonly used and includes three groups according to the site of origin and the vessel course.^[5]^ Single coronary artery has a low prevalence in general population ranging from 0.014% to 0.066%.^[5,6]^

Coronary congenital absence is thought to be caused by a defect of coronary development during the embryonic period resulting in a coronary artery anomaly^[1]^ and it may be also associated with other congenital heart disease.^[3]^ This condition has to be differentiated by congenital ostial atresia, which represents a separate entity characterized by partial or total arterial orifice occlusion and corresponding proximal coronary hypoplasia.^[7,8]^

Patients may be asymptomatic or present with myocardial ischemia, acute coronary syndrome, syncope, ventricular fibrillation, or sudden death.^[6]^ Many authors^[9,10]^ consider some mechanisms explaining myocardial ischemia in single coronary artery, such as the coronary steal phenomenon due to the abnormal vessel or microvascular damage, and slow controlled ischemia caused by long travel distance of abnormal coronary artery. Shirani et al^[11]^ reported that 15% of patients with a single coronary artery had myocardial ischemia due to the direct consequence of the coronary anomaly.

As for ECG alterations subsequent to single coronary artery, Yan et al^[6]^ reported that the ECG manifestations of a patient with single left coronary artery may vary from no changes to various findings such as nonspecific ST-T wave changes to supraventricular arrhythmia. To a certain extent this may be explained by the blood supply to sinoatrial (SA) and atrioventricular (AV) node. While RCA normally supply these nodes, it is done so by a single left coronary artery and its branches. Lack of adequate blood supply from this anomaly may lead to ischemia in SA and AV node with eventual fibrosis and dysfunction which might be manifested in ECG varying from normal to abnormal readings related to ischemia or arrhythmia.

The relationship between the clinical manifestations and congenital absence of the RCA is still unknown. It is presumed that during an early age, absence of risk factors such as atherosclerosis, hypertension and diabetes may be the reason behind the lack of clinical symptoms. Patients are symptomatic only when the disease of coronary artery begins to occur.

Multidetector computed tomography has gained a crucial role in cardiovascular disease diagnosis and the recent technological improvements have widened its application allowing more patients to be acquired with contextual radiation dose reduction.^[12]^ The updated 2016 NICE guideline indicates CTCA as the first-line investigation in all patients with atypical or typical angina symptoms or those who are asymptomatic with suggested ECG changes for ischemia.^[13]^ At the same time, CTCA is increasingly applied in congenital heart disease diagnosis and follow-up providing an excellent visualization of systemic venous, abnormalities, extracardiac blood vessels, and coronary arteries.^[14]^ Invasive coronary angiography is considered the gold standard for the diagnosis and treatment of coronary diseases even if it shows the vessel lumen not providing information about the vessel wall or the surrounding structures.^[1]^ Moreover, when a coronary absence occurs, the operator is faced with a more laborious procedure when trying to catheterize a non-present coronary ostium.^[6]^

In our case, the results of CTCA clearly demonstrate that myocardial ischemia is not provoked by coronary artery disease. Moreover, CTCA revealed RCA absence and displayed a markedly dominant LCX giving off branches to the right atrium and the right ventricle (Group I Lipton anomalies, L-I pattern). Even though this can be considered a potentially serious coronary anomaly,^[4]^ CTCA underlined the absence of angina related features such as acute angle take-off, slit-like ostium, ostial tissue flaps, coronary intussusception, or aberrant course between the aorta and the pulmonary artery.

As for patient management, currently, there is no standardized procedure or guideline from the evidence-based medicine for the treatment of congenital absence of the RCA. Choice of treatment may include either conservative treatment with anti-platelets, lipid-lowering, anti-hypertensive therapy, etc or with interventional therapy such as coronary artery revascularization, pacemaker implantation and other cardiac surgical procedures.^[6]^

Angelini^[15]^ investigated coronary artery anomalies and proposed a diagnostic protocol for adult patients who are at risk for coronary artery anomalies where percutaneous transluminal coronary angioplasty or surgery are considered only in case of severe narrowing due to intramural course. Asymptomatic right anomalous origin of the coronary artery arising from the opposite sinus is even more challenging, given its weaker association with sudden cardiac death. In a significant number of asymptomatic cases, intervention may not be warranted, particularly if there is no evidence of ischemia in the patient.^[16]^

In conclusion, CTCA confirmed to be the choice technique for coronary visualization and CAD exclusion, reducing the number of inconclusive invasive procedures.

## Author contributions

**Conceptualization:** Ernesto Forte.

**Resources:** Ernesto Forte.

**Supervision:** Carlo Cavaliere.

**Visualization:** Bruna Punzo, Marco Agrusta, Marco Salvatore, Gianluca Spidalieri.

**Writing – original draft:** Ernesto Forte.

**Writing – review & editing:** Ernesto Forte, Bruna Punzo.
